# Mechanistic and applied study of phosphofructokinases, the “gatekeeper” of the glycolytic pathway on the central carbon metabolism

**DOI:** 10.1016/j.mec.2025.e00268

**Published:** 2025-12-24

**Authors:** Lingyun Li, Xin Chen, Yijie Zhang, Ning Qin, Yu Chen, Xu Ji, Jens Nielsen, Zihe Liu

**Affiliations:** a College of Life Science and Technology, Beijing Advanced Innovation Center for Soft Matter Science and Engineering, Beijing University of Chemical Technology, 100029, Beijing, China; b Department of Life Sciences, Chalmers University of Technology, SE412 96, Gothenburg, Sweden; c CAS Key Laboratory of Quantitative Engineering Biology, Shenzhen Institute of Synthetic Biology, Shenzhen Institute of Advanced Technology, Chinese Academy of Sciences, Shenzhen, 518055, China; d BioInnovation Institute, Ole Maaløes Vej 3, DK2200, Copenhagen N, Denmark

**Keywords:** *PFK1*, *PFK2*, Glycolysis, Oxidative respiration, Free fatty acid, Yeast cell factories

## Abstract

Phosphofructokinase (Pfk), a key regulatory enzyme in glycolysis, is composed of Pfk1 and Pfk2 subunits in *Saccharomyces cerevisiae*. However, the distinct roles of these subunits in central carbon metabolism remain unclear. Here, we examined the metabolic consequences of deleting *PFK1* or *PFK2*. The *pfk2Δ* strain exhibited more severe defects than *pfk1Δ*. Its maximum specific growth rate was reduced by approximately 54 % in *pfk2Δ* and by about 15 % in *pfk1Δ*, both relative to the reference strain. Ethanol production decreased by 36 % and 82 % in *pfk1Δ* strain and *pfk2Δ* strain, respectively, relative to the reference strain. Both deletion strains accumulated higher acetate levels compared to the reference strain, increasing by 25.4 % in the *pfk1Δ* strain and 82 % in the *pfk2Δ* strain. Flux balance analysis (FBA) revealed a markedly increased carbon flux to the tricarboxylic acid cycle (TCA) in the *pfk2Δ* strain, with respiration-associated carbon flux elevated 1.5-fold compared to the *pfk1Δ* strain. Consistently, transcriptomic profiling showed significant upregulation of respiration-related genes in the *pfk2Δ* strain compared to the reference strain. Notably, deletion of *PFK2* enhanced acetyl-CoA-derived product formation, with free fatty acid (FFA) titers increasing from 412 mg L^−1^ to 517 mg L^−1^ (a 33.3 % increase). These findings establish *PFK2* as a key regulatory node redirecting carbon flux from fermentation toward respiration and biosynthesis, offering new opportunities for metabolic engineering of acetyl-CoA-derived products.

## Highlights

• *PFK1* and *PFK2* play distinct regulatory roles in yeast central carbon metabolism, differentially affecting growth and fermentation.
• *PFK2* deletion redirects carbon flux toward mitochondria, enhancing oxidative respiration and energy production.
• *PFK2* deletion increases free fatty acid production, highlighting its potential as a metabolic engineering target for cell factories.

## 1 Introduction

Glycolysis is a highly conserved metabolic pathway in all three kingdoms, whose main function is to break down glucose into pyruvate and generate energy in the form of ATP (Alam et al., 2023; Cool et al., 2023; Shen et al., 2024) and NADH (Flamholz et al., 2013). Phosphofructokinase (Pfk) catalyzes the phosphorylation of fructose-6-phosphate using ATP to produce fructose-1,6-bisphosphate. It is generally considered to be a key flux controlling enzyme in glycolysis and is regulated by multiple allosteric factors (Qin et al., 2023; Compton and Patrick, 2025), including activation by fructose-2,6-bisphosphate (F2,6BP) (Heinisch et al., 1996) and AMP(Bañuelos et al., 1977), and inhibition by ATP (Rodicio et al., 2000) and citrate (Martínez-Costa et al., 2012). Mutagenesis of these regulatory sites (Heinisch et al., 1996; Rodicio et al., 2000) has revealed their importance in maintaining glycolytic flux, growth on different carbon sources, and cell wall integrity, underscoring the essential role of allosteric control in coordinating metabolic balance. In *Saccharomyces cerevisiae*, Pfk is an octameric structure consisting of four α subunits and four β subunits, encoded by *PFK1* and *PFK2*, respectively (Boonekamp et al., 2022). *PFK1* is located on chromosome VII, while *PFK2* is located on chromosome XIII. Amino acid sequence alignment shows that: (i) the N-terminal and C-terminal regions of each subunit have approximately 20 % homology; (ii) there is approximately 55 % sequence homology between the α and β subunits; (iii) the Pfk proteins also show significant conservation with mammals (such as human and rabbit muscle, 42 %), *Escherichia coli* (34 %), and *Bacillus* (36 %) (Heillisch et al., 1989).

Early biochemical studies suggested that Pfk1 is mainly responsible for allosteric regulation, whereas Pfk2 exerts stronger catalytic activity (Tijane et al., 1980). However, subsequent mutagenesis and kinetic analyses demonstrated that both Pfk1 and Pfk2 contribute to catalytic and regulatory control (Arvanitidis and Heinisch, 1994; Heinisch et al., 1996). Beyond its role in glycolysis, Pfk2 has been implicated in several moonlighting functions, including glucose-induced V-ATPase reassembly and vacuolar pH regulation (Chan et al., 2016; Chan and Parra, 2014), RNA binding and ATP-dependent RNA unwinding that modulate translation, and participation in RNA-rich G-bodies (Albihlal et al., 2025). These non-glycolytic roles highlight Pfk2 as a multifunctional integrator of metabolic, pH-regulatory, and translational processes. However, how the loss of Pfk1 or Pfk2 influences central carbon redistribution and global gene expression remains poorly understood.

Some studies on the physiology of *PFK1* and *PFK2* have found, although single *PFK* gene mutant can synthesize a small amount of ethanol under glucose conditions (Zheng et al., 2024), there is a delay of about 2 h in ethanol production when switching from a non-fermentable carbon source to glucose, while control cells can immediately initiate ethanol fermentation (Breitenbach-Schmitt et al., 1984b). When yeast carrying a single *PFK* gene mutation that results in the loss of Pfk activity is shifted from a medium with a non-fermentable carbon source to one containing glucose and antimycin A (a respiratory inhibitor), it is unable to grow. If antimycin A is added after the cells have adapted to glucose, they are able to continue growing (Breitenbach-Schmitt et al., 1984a). However, *PFK1* and *PFK2* double knockout strain completely loses its ability to synthesize ethanol (Qin et al., 2023), suggesting that the activity of this enzyme is essential for fermentative metabolism (Qin et al., 2023). Current research mainly focuses on the changes in metabolic phenotypes caused by the *PFK* genes, while mechanistic insights into how Pfk influences central carbon metabolism remain limited. In this study, we systematically analyzed the roles of *PFK1* and *PFK2* in regulating central metabolism in *S. cerevisiae*. Our findings enhance the understanding of the regulatory interplay between fermentation and respiration in *S. cerevisiae*, which is critical for metabolic engineering applications.

## 2 Results

### 2.1 Physiological impact upon *pfk1Δ* strain and *pfk2Δ* strain

To elucidate the contributions of *PFK1* and *PFK2* to central carbon metabolism in *S. cerevisiae*, we carried out aerobic, glucose-fed batch fermentations in *pfk1Δ* and *pfk2Δ* strains, respectively. The *pfk1Δ* and *pfk2Δ* strains exhibited maximal specific growth rates (μ_max_) of 0.35 h^−1^ and 0.19 h^−1^, respectively, compared with 0.41 h^−1^ for the reference strain (Supplemental Fig. 1, Table 1). Correspondingly, specific glucose uptake rates declined by 35.1 % in *pfk1Δ* strain and by 74.3 % in *pfk2Δ* strain relative to the reference strain. Ethanol production rates were reduced by 36 % in the *pfk1Δ* strain and by 82 % in the *pfk2Δ* strain, while acetate production increased by 25.4 % and 82 %, respectively (Table 1). Because the *pfk* mutants displayed markedly reduced growth rates, all metabolic rates were normalized to the specific growth rate to better compare metabolic efficiency among strains. After normalization, ethanol formation decreased by 17.4 % in *pfk1Δ* and by 60.4 % in *pfk2Δ* relative to the reference strain (Supplementary Table 1), indicating that *PFK2* deletion imposes a substantially stronger constraint on fermentative carbon flux. These findings indicate that loss of *PFK2* function causes a more pronounced disruption of glycolytic flux than the deletion of *PFK1*. We therefore hypothesize that, to offset compromised ATP generation via glycolysis, carbon flux in the deletion strains is rerouted toward mitochondrial respiration.

**Table 1:** Overview of physiological parameters for strains.

	Reference strain	*pfk1Δ*	*pfk2Δ*
Specific growth rate	0.41 ± 0.0057	0.35 ± 0.0023∗∗∗	0.19 ± 0.0011∗∗∗
*q*_Glucose_ (mmol g_DW_^−1^h^−1^)	−19.1 ± 1.81	−12.4 ± 0.14∗	−4.9 ± 0.2∗∗
*q*_Ethanol_ (mmol g_DW_^−1^h^−1^)	28.5 ± 0.17	18.24 ± 0.2∗∗∗	5.23 ± 0.25∗∗∗
*q*_Acetate_ (mmol g_DW_^−1^h^−1^)	0.35 ± 0.023	0.44 ± 0.011∗∗∗	0.63 ± 0.0090∗∗
*q*_Glycerol_ (mmol g_DW_^−1^h^−1^)	2.7 ± 0.045	0.99 ± 0.0136∗∗∗	0.22 ± 0.0125∗∗∗
*P*_Biomass_ (g L^−1^h^−1^)	0.34 ± 0.0047	0.27 ± 0.0017∗∗∗	0.16 ± 0.0010∗∗∗
*P*_Ethanol_ (g L^−1^h^−1^)	0.98 ± 0.26	0.347 ± 0.0023∗	0.198 ± 0.0124∗∗

Cultivations were carried out in minimal medium containing 2 % (w/v) glucose under aerobic conditions. Dissolved oxygen (DO) was controlled at > 30 % air saturation by adjusting stirring speed and aeration rate. Values represent mean ± 95 % confidence intervals calculated from three biological replicates (n = 3). Statistical significance was assessed using a two-tailed unpaired Student's *t*-test (∗*p* < 0.05, ∗∗*p* < 0.01, ∗∗∗*p* < 0.001, n.s., not significant).

### 2.2 Flux balance analysis (FBA) of *pfk1Δ* and *pfk2Δ* strains

**Fig. 1:**
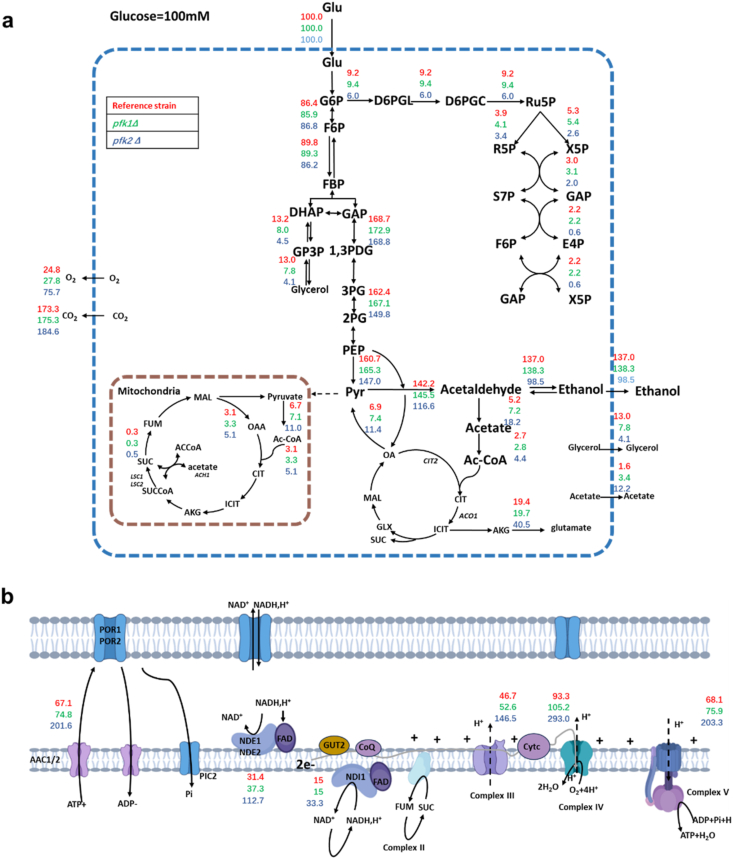
**FBA analysis of *pfk1Δ* and *pfk2Δ* strains.** FBA prediction of metabolic flux distributions in central metabolic pathways (a) and electron transport chain (b) from reference strain, *pfk1Δ* strain and *pfk2Δ* strain during the log phase of the batch culture. Fluxes were normalized to 100 units of glucose uptake in central metabolic pathways. Glu: glucose; G6P: glucose-6-phosphate; F6P: fructose-6-phosphate; FBP: fructose-1,6-phosphate; DHAP: dihydroxyacetone phosphate; GAP: glyceraldehydes-3-phosphate; GP3P: glyceraldehyde 3-phosphate; 1,3PDG: 1,3-diphosphoglycerate; 3 PG: 3-phosphoglycerate; 2 PG: 2-phosphoglycerate; PEP: phosphoenolpyruvate; Pyr: pyruvate; D6PGL: 6-phosphogluconolactonase; D6PGC: 6-phosphogluconate dehydrogenase; Ru5P: ribulose-5-phosphate; R5P: ribose-5-phosphate; X5P: xylulose-5-phosphate; S7P: sedoheptulose-7-phosphate; E4P: erythrose-4-phosphate; OA: oxaloacetate; MAL: malate; GLX: glyoxylate; SUC: succinate; ICIT: isocitrate; AKG: alpha-Ketoglutarate; CIT: citrate; FUM: fumarate.

To validate our hypothesis, we performed flux balance analysis (FBA) to quantify intracellular carbon fluxes in the deletion strains. Compared to the reference strain, the *pfk1Δ* strain exhibited a 39.3 % decrease in the glycerol production flux, a 38.5 % increase in acetate production flux, a modest 6 % enhancement of TCA cycle flux. Perturbations were more pronounced in the *pfk2Δ* strain: glycerol production flux fell by 68.5 %, acetate production flux surged by 250 %, ethanol production flux declined by 27.9 %, and the TCA cycle flux rose by 64.2 % (Fig. 1a).

The yeast electron-transport chain (ETC) comprises two NADH dehydrogenases, Nde1 and Ndi1(Yu et al., 2022), which are functionally similar to mammalian Complex I. These are followed by Complexes III and IV, which generate the proton-motive force required for ATP synthesis via Complex V (Das et al., 2021). In the *pfk1Δ* strain, carbon flux through Nde1/Ndi1 increased by 18.8 %, Complex III by 12.6 %, Complex IV by 12.8 %, and Complex V by 11.5 %, compared to the reference strain. In contrast, the *pfk2Δ* strain displayed much larger flux increase: 259 % through Nde1/Ndi1, 214 % through Complex III, 214 % through Complex IV, and 199 % through Complex V (Fig. 1b). FBA results indicate that deletion of either *PFK1* or *PFK2* shifts carbon flux toward the tricarboxylic acid (TCA) cycle and the mitochondrial electron transport chain, thereby enhancing respiratory activity. Among the two, *PFK2* deletion exerts a more pronounced effect on this metabolic reprogramming compared to *PFK1* deletion.

### 2.3 Transcriptional profiles of *pfk1Δ* and *pfk2Δ* strains

**Fig. 2:**
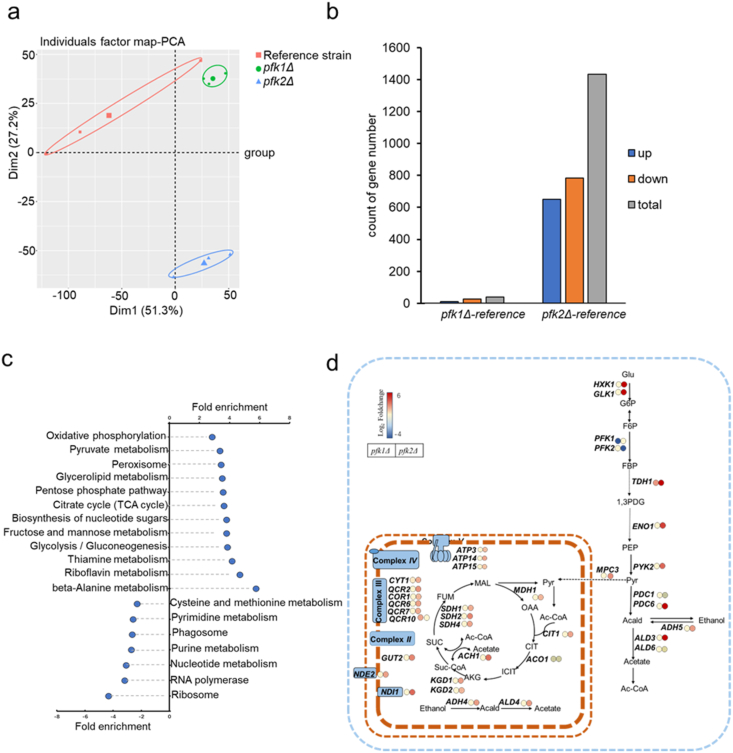
**Transcriptional responses in *pfk1Δ* and *pfk2Δ* strains.** (a) PCA of the gene expression in reference strain, *pfk1****Δ*** and *pfk2****Δ*** strains. (b) Significantly differentially expressed genes number (*p*-adj <0.05 (Benjamini-Hochberg method)) in the *pfk1Δ* and *pfk2Δ* strains compared with the reference strain. (c) Gene set enrichment analysis of significantly differentially expressed genes in *pfk2Δ* strains compared with the reference strain (KEGG pathway, FDR <0.05). (d) Fold changes in the expression of genes that encode central metabolic pathways in *pfk1Δ* and *pfk2Δ* strains compared with the reference strain. Fold enrichment indicated the magnitude of enrichment against the genome background of the strain S288C analyzed via DAVID.

To elucidate the mechanisms underlying the altered metabolic flux in the *PFK* knockout strains, we performed RNA-seq analyses on *pfk1Δ* and *pfk2Δ* strains. RNA-seq analysis revealed that *PFK1* and *PFK2* transcript levels remained largely unchanged in the reciprocal deletion strains (*PFK1* in *pfk2Δ*: log_2_ fold change = −0.1018, *p*-adj = 0.665; *PFK2* in *pfk1Δ*: log_2_ fold change = −0.0955, *p*-adj = 0.83), indicating that transcriptional compensation between the two genes is minimal. Principal component analysis (PCA) demonstrated strong grouping of biological replicates. The transcriptome of the *pfk2Δ* strain diverged significantly from the reference strain and the *pfk1Δ* strain (Fig. 2a). Differential expression analysis identified that the expression of 40 genes in *pfk1Δ* strain and 1432 genes in *pfk2Δ* strain was significantly different (*p*-adj <0.05, log_2_FC > 1) compared to the reference strain (Fig. 2b). Among them, 31 genes were commonly affected in both knockouts. The number of differentially expressed genes (DEGs) in *pfk1Δ* strain was much smaller than the *pfk2Δ* strain, suggesting a relatively limited transcriptional response.

Gene Ontology (GO) enrichment analysis of DEGs revealed that no significantly changed metabolic pathways in the *pfk1Δ* strain compared to the reference strain. In contrast, *pfk2Δ* strain showed pronounced upregulation of gene sets associated with central carbohydrate metabolism, including gluconeogenesis, pentose phosphate pathway, TCA cycle, and oxidative phosphorylation (Fig. 2c), which were consistent with both our FBA flux data and Physiological data (Fig. 1a and b, Table 1). Moreover, genes involved in mitochondrial electron transport and cellular respiration were also significantly upregulated in the *pfk2Δ* strain (Supplemental Fig. 2), indicating a broader metabolic adjustment and a stronger transcriptional response to *PFK2* deletion. These findings are consistent with previous reports showing that Pfk1 primarily contributes to allosteric regulation while retaining catalytic activity (Heinisch et al., 1996), whereas Pfk2 provides the major catalytic function (Tijane et al., 1980), thereby influencing carbon flux redistribution upon *PFK2* deletion.

To delineate how *PFK1* and *PFK2* deletions affect central carbohydrate metabolism, we examined the expression levels of key glycolytic and respiratory genes (Fig. 2d). Compared to the reference strain, there were few differentially expressed genes related to central metabolism in the *pfk1Δ* strain. In contrast, *pfk2Δ* strain exhibited broad transcriptional activation of both glycolytic and respiratory pathways. Notably, several glycolytic genes, including *HXK1* and *GLK1*, were upregulated in the *pfk2Δ* strain. This upregulation may indicate a compensatory mechanism to enhance glucose uptake, potentially offsetting the reduced glycolytic flux resulting from *PFK2* deletion. This observation underscores the value of integrating transcriptomic and metabolic analyses to better understand carbon flux redistribution. Furthermore, multiple genes associated with the tricarboxylic acid (TCA) cycle were upregulated, including *MPC3* (mitochondrial pyruvate carrier), *MDH1* (mitochondrial malate dehydrogenase), and *KGD1* and *KGD2* (subunits of mitochondrial alpha-ketoglutarate dehydrogenase) (Fig. 2d). Additionally, genes involved in the mitochondrial respiratory chain were also upregulated, such as *NDI1* and *NDE2*, along with components of complexes II, III, IV, and ATP synthase (Fig. 2d). These transcriptional changes are consistent with the flux balance analysis (FBA) results (Fig. 1). Collectively indicating that *pfk2Δ* reprograms central carbon metabolism toward enhanced respiratory capacity and potential metabolic compensation upstream of glycolysis.

### 2.4 Effects of the deletion of *PFK1* and *PFK2* on the respiro-fermentative metabolism

**Fig. 3:**
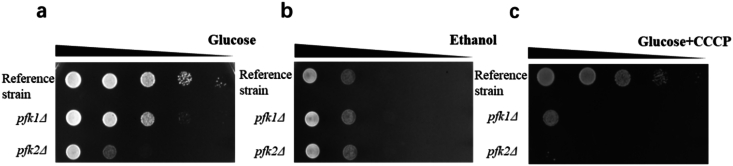
**Effects of the deletion of *PFK1* and *PFK2* on respire-fermentative metabolism.** Spotting assay of the reference strain, *pfk1Δ* and *pfk2Δ* strains on plates with glucose (a), ethanol (b) and glucose + CCCP (c) as the carbon source, respectively. Spots from a representative experiment are shown, similar results were obtained from 3 biological replicates.

To examine the balance between respiration and fermentation, we further evaluated cell growth on ethanol as the sole carbon source. Although *pfk1Δ* and *pfk2Δ* grew less than the reference strain on glucose (Fig. 3a), they grew equally well better than the reference strain when ethanol was used as the sole carbon source (Fig. 3b), which suggest that the deficiency in growth on glucose may be explained by reduced fermentative capacity, with equal or perhaps enhanced respiratory ability. To further validate this shift, we treated strains with 10 μM carbonyl cyanide 3-chlorophenylhydrazone (CCCP) which is an uncoupler that collapses the mitochondrial proton gradient and inhibits oxidative phosphorylation (Qin et al., 2023). Under CCCP stress, both deletion mutants exhibited markedly slower growth compared to the reference strain, with *pfk2Δ* strain being the most sensitive one (Fig. 3c). Taken together, these data demonstrate that loss of *PFK2* function diminishes the flux to fermentation, forcing cells to rely on respiration for energy, which in turn enhances mitochondrial flux and substrate-use efficiency.

### 2.5 Effects of the deletion of *PFK1* or *PFK2* on the fatty acid production

*S. cerevisiae* is widely used in both research and industry for the production of chemicals, fuels, and pharmaceutical (Qin et al., 2024; Li et al., 2025; Yilmaz et al., 2022). However, ethanol accumulation remains a major by-product during biosynthesis, and enhancing the efficiency of cellular energy utilization remains a significant challenge (Nielsen, 2019). Our results showed that the deletion of either *PFK1* or *PFK2* reduced ethanol formation, reshaped central carbon flux toward respiration, and boosted respiratory activity, indicating that phosphofructokinase is a valuable engineering target for improving yeast cell factories. The observed accumulation of acetate (Table 1) in *pfk* mutants likely reflects altered cytosolic acetyl-CoA homeostasis, which may increase the precursor pool available for fatty acid biosynthesis. Because free fatty acids (FFAs) are important chemical feedstocks and potential biofuels, we applied *PFK1* and *PFK2* deletions to FFA-producing *S. cerevisiae* strains.

**Fig. 4:**
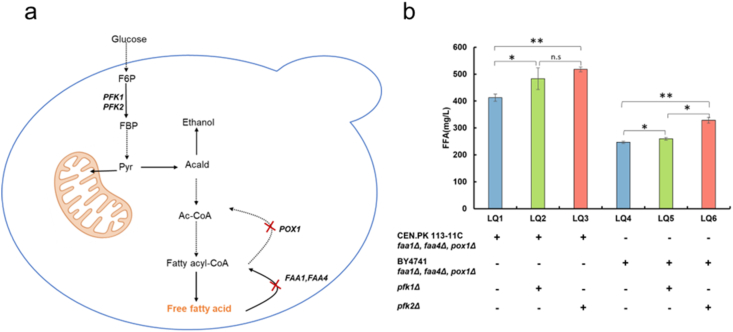
**Effects of the deletion of *PFK1* or *PFK2* on production of free fatty acid.** (a) Free fatty acid synthesis pathway. (b) Free fatty acid production in *pfk1Δ* or *pfk2Δ* strains. The data represents three biological replicates, and the error bars represent standard deviations. ∗*p* < 0.05, ∗∗*p* < 0.01, n.s., not significant compared to the reference strain.

First, in the *S. cerevisiae* CEN.PK*113-11C* strain, we deleted the fatty acyl-CoA synthetase genes *FAA1* and *FAA4*, along with the acyl-CoA oxidase gene *POX1* (Fig. 4a) (Zhou et al., 2016) to block free fatty acid (FFA) oxidation, resulting in an FFA titer of 412.2 mg L^−1^. Subsequent knockout of *PFK1* or *PFK2* led to increases in FFA titers by 17.2 % and 25.4 %, respectively, compared to the reference strain (Fig. 4b). To determine whether this effect was generalizable, we further evaluated the effects of *PFK1* and *PFK2* on FFA production in the BY4741 background strain with triple deletion of *FAA1*, *FAA4*, and *POX1*. In this context, the deletion of *PFK1* yielded a 5.3 % increase in FFA production (*p* < 0.05. Fig. 4b), whereas *PFK2* deletion boosted FFA levels by 33.3 % relative to the reference strain (*p* < 0.01. Fig. 4b). Moreover, the results show that in the CEN.PK background, the FFA titer of the *pfk2Δ* strain was not significantly different from that of the *pfk1Δ* strain. In contrast, in the BY4741 background, the *pfk2Δ* strain exhibited a significantly higher FFA titer than *pfk1Δ*, highlighting a stronger impact of *PFK2* deletion on fatty-acid overproduction in this genetic background. These results demonstrate that reducing Pfk activity can redirect carbon toward acetyl-CoA and its downstream lipid products across two different yeast strains. The knockout of *PFK2* offers a novel strategy for enhancing the production of acetyl-CoA-derived bioproducts.

## 3 Discussion

*S*. *cerevisiae* is a well-known Crabtree-positive yeast that preferentially utilizes fermentation over respiration for energy production, even in the presence of oxygen (Pronk et al., 1996; De Deken, 1966; Dai et al., 2018a; Zhang et al., 2015; Fiechter and Seghezzi, n.d.). Although fermentation yields only two ATP molecules per glucose, compared to approximately 16 from respiration (Bakker et al., 2001), this metabolic preference is widely observed. Several hypotheses have been proposed to explain the Crabtree effect. Early studies attributed the phenomenon to the higher efficiency of glycolysis in terms of ATP production per unit mass of protein (Flamholz et al., 2013), while more recent research suggests that elevated expression of glycolytic enzymes diverts carbon flux toward fermentative metabolism (Shen et al., 2024). Among the key enzymes in glycolysis, phosphofructokinase, composed of the subunits Pfk1 and Pfk2-catalyzes one of the few irreversible reactions (Boles et al., 1993; Gupta and Gupta, 2021), positioning it as a critical control point in the balance between fermentation and respiration. Accordingly, attenuation or deletion of *PFK1/2* reduces glycolytic capacity and ATP generation, thereby relieving glucose repression and promoting respiratory metabolism (Fig. 1, Fig. 2d). This shift toward increased respiration upon *PFK* ablation is consistent with the classical understanding of the Crabtree effect. In line with this observation, the higher biomass yield observed (Supplemental Table 1) in the *PFK2* deletion strains may also reflect enhanced respiratory energy metabolism. Increased activity of ATP synthase and NADH dehydrogenases could improve ATP generation through oxidative phosphorylation, thereby supporting cell growth at the expense of overflow metabolite synthesis.

While previous studies have reported reduced ethanol production following *PFK* deletion (Breitenbach-Schmitt et al., 1984b; Qin et al., 2023), the differential contributions of *PFK1* and *PFK2* had not been systematically compared. In this study, we dissected their individual roles and found that *pfk2Δ* strain exerted a more pronounced impact on central carbon metabolism than *pfk1Δ* strain, as revealed by flux balance analysis (FBA) and transcriptomic profiling. While the overall flux patterns predicted by the FBA aligned well with the experimental data, some discrepancies were evident, for example, the model predicted no change in ethanol flux in the *pfk1Δ* strain, whereas experiments showed an approximately 17.4 % decrease (Supplemental Table 1). These differences likely arise from the simplified assumptions of constraint-based modeling, but omits regulatory, kinetic, and redox limitations that influence flux realization in vivo. The FBA simulations were constrained by experimentally measured growth and substrate uptake rates. Therefore, the model outputs are not independent predictions but rather serve to rationalize the observed redistribution of carbon flux in *pfk1Δ* and *pfk2Δ*. While such constraint-based modeling provides a valuable system-level view of feasible metabolic states, its predictive capacity is inherently limited by the physiological data used for constraint and the steady-state optimization assumptions.

Although the *pfk* deletion strains exhibited slower specific growth rates, their overall biomass accumulation under batch culture remained comparable to that of the reference strain (Supplemental Table 1). Notably, the *pfk2Δ* strain even achieved a higher final biomass than the reference strain, suggesting improved carbon-use efficiency. This observation is consistent with the reduced carbon loss to ethanol and glycerol formation (Table 1) and highlights the potential of *PFK2* modulation to enhance fatty acid biosynthesis in engineered yeast strains. Building on these insights, we further evaluated the metabolic engineering potential of *PFK* modulation. We introduced *PFK1* and *PFK2* deletions into a free fatty acid (FFA)-producing yeast strain, in which acetyl-CoA serves as a key precursor. Both deletions led to enhanced FFA production, with the *pfk2Δ* strain achieving a 33 % increase (Fig. 4b). These findings highlight the potential of *PFK* modulation not only in suppressing ethanol overflow but also in boosting the biosynthesis of acetyl-CoA-derived products. Consistent with this interpretation, carbon rerouting through the pentose phosphate pathway (PPP) and the pyruvate dehydrogenase (PDH) bypass likely supports growth of the *pfk2Δ* strain despite reduced phosphofructokinase activity (Fig. 2c). Accumulation of glucose-6-phosphate (G6P) and fructose-6-phosphate (F6P) would be expected to channel carbon into the oxidative PPP, generating erythrose-4-phosphate (E4P) and glyceraldehydes-3-phosphate (GAP) via transaldolase and transketolase reactions that feed into lower glycolysis and the TCA cycle (Fig. 1, Fig. 2c), At the same time, diminished ethanol overflow may redirect carbon through the PDH bypass (pyruvate decarboxylase (Pdc) → aldehyde dehydrogenase (Ald6) → Acetyl-coA synthetase (Acs)), enhancing cytosolic acetyl-CoA and NADPH production via Ald6. This carbon-rerouting strategy provides a compensatory supply of biosynthetic precursors and redox cofactors, albeit at the expense of glycolytic efficiency, thereby explaining the slower growth and increased acetate and lipid accumulation observed in the *pfk2Δ* strain. Given acetyl-CoA's central role in the production of isoprenoids (Li et al., 2024), polyketides (Sha et al., 2025), and polyhydroxyalkanoates (Bhola et al., 2021), the metabolic rewiring demonstrated here could be extended to various high-value biosynthetic pathways.

The stronger metabolic perturbation caused by *pfk2Δ* strain may be attributed to regulatory differences between the two Pfk subunits. Possible mechanisms include divergent expression profiles under glucose-rich conditions, distinct post-translational modifications, or specific interactions with key metabolic regulators (Chan et al., 2016; Qiao et al., 2024; Tijane et al., 1980), Although these hypotheses remain to be experimentally validated, they underscore the complexity of Pfk regulation and suggest that Pfk2 may play a more dominant role in coordinating glycolytic flux and maintaining energy homeostasis under fermentative conditions. In addition to these regulatory differences, Pfk2's moonlighting activities could also contribute to the distinct physiological phenotypes observed in the *pfk2Δ* strain. Loss of Pfk2 may disrupt glucose-triggered V-ATPase reassembly and vacuolar pH regulation, thereby altering intracellular pH and metabolic signaling. In addition, Pfk2's RNA-binding and ATP-dependent RNA-unwinding activities may modulate the expression of genes involved in carbon metabolism and lipid biosynthesis. Disruption of these noncanonical functions may therefore underlie the reduced growth and ethanol production, as well as the increased accumulation of free fatty acids, observed in *pfk2Δ* cells. To further test the functional contribution of Pfk2, we introduced an additional copy of *PFK1* under the control of the native *PFK2* promoter and terminator in the *pfk2Δ* background. This complementation partially rescued the growth defect, leading to a measurable increase in specific growth rate, although the strain did not fully recover to the reference level (Supplementary Fig. 3). Because this design equalizes the transcriptional context between the complemented *PFK1* and the deleted *PFK2* allele, the partial rescue indicates that Pfk1 cannot fully substitute for Pfk2's noncanonical regulatory functions. Together, these findings support the view that Pfk2 serves as a multifunctional integrator linking glycolytic flux, pH regulation, and translational control to sustain carbon and energy balance.

Beyond Pfk, other glycolytic nodes have also been targeted for flux redirection. For example, deletion or repression of *PGI1* can shift flux toward the oxidative pentose phosphate pathway, increasing NADPH availability for biosynthesis (Fiaux et al., 2003), down-regulating pyruvate kinase (*PYK*) activity reduces glycolytic flux and favors respiration (Yu et al., 2018), while attenuation of pyruvate decarboxylase (*PDC*) (Dai et al., 2018b; Yu et al., 2018), or enhancement of pyruvate dehydrogenase complex (*PDH*) (Zhang et al., 2020) directs more carbon into mitochondrial acetyl-CoA production rather than ethanol. These complementary interventions suggest that combinatorial engineering of *PFK*, *PYK*, *PDC*, and *PDH* could provide fine control over carbon flux and enhance precursor availability for diverse biosynthetic goals. However, complete deletion of *PFK* significantly impairs cell growth on glucose, presenting a major barrier for industrial applications. To overcome this constraint, adaptive laboratory evolution (ALE) can be employed to identify compensatory mutations that restore growth while preserving the desired metabolic rewiring. For example, Zhang et al. (2015) demonstrated that *PDC*-deficient yeast strains evolved mutations in *MTH1*, a negative regulator of glucose uptake. These mutations restored growth by reducing glucose influx and alleviating fermentative stress. Given that Pfk acts at an early stage of glycolysis, its deletion may similarly downregulate glucose uptake indirectly. Therefore, combining *PFK* and *PDC* deletions, possibly together with glucose transporter modulation, could synergistically suppress overflow metabolism and redirect flux toward biosynthesis.

In addition to ALE, synthetic regulatory systems such as inducible promoters, metabolite-responsive circuits, and CRISPR-based gene switches offer opportunities to dynamically modulate glycolytic flux in response to environmental or intracellular signals. Such dynamic control may enable better trade-offs between growth and production, especially in industrial fermentation settings. Moving forward, a system-level understanding of how yeast cells adapt to *PFK* attenuation will be essential for designing robust strains. Furthermore, integrating transcriptomic or proteomic data into enzyme-constrained frameworks such as ecYeastGEM/GECKO could further elucidate the mechanistic basis of *PFK*-mediated flux regulation in fatty acid–producing yeast strains. Together, these insights lay the groundwork for engineering high-performance yeast cell factories for the sustainable production of acetyl-CoA–derived and other value-added compounds.

In conclusion our integrated physiological, transcriptomic, and flux-balance analyses reveal that Pfk1 exerts a modest regulatory influence on central carbon metabolism in *S. cerevisiae,* while Pfk2 appears to play a central regulatory role, helping to balance fermentative glycolysis and oxidative respiration, thereby coordinating energy and biosynthetic networks in yeast. Fine-tuning the expression of *PFK2* can serve as a powerful strategy for better understanding of energy metabolism and for rational engineering of yeast cell factories for enhanced lipid production.

## 4 Materials and methods

### 4.1 Strain and materials

*The S. cerevisiae strain* CEN.PK *113-7D* served as the host for constructing the *pfk1Δ* and *pfk2Δ* strains. The *kanMX* cassette was PCR-amplified from plasmid pUG 6 and utilized for individual gene deletions of *PFK1* or *PFK2* via homologous recombination. For the free fatty acid-producing strains constructed, the CEN.PK *113-11C* and BY4741 were used as background strains, respectively, and the GTR-CRISPR system (Zhang et al., 2019) was employed for the deletion of *PFK1*, *PFK2*, *FAA1*, *FAA4* and *POX1*. The strains and plasmids used in this study are summarized in Tables S1 and 2, and the primers used are summarized in Table S3.

### 4.2 Shake flasks cultivation

Selection of the CEN.PK *113-7D pfk1::kanMX* and CEN.PK *113-7D pfk2::kanMX* strains was performed on yeast extract peptone dextrose (YPD) plates supplemented with 600 mg L ^−1^ G418 (Sinopharm, Beijing, China). The growth test in shake flasks and bioreactor was carried out using a defined minimal medium (Verduyn et al., 1992), consisting of 7.5 g L^−1^ (NH_4_)_2_SO_4_, 14.4 g L^−1^ KH_2_PO_4_, 0.5 g L^−1^ MgSO_4_·7H_2_O and 2 % glucose. The pH of the medium was adjusted to 6.5 using KOH. After autoclaving the medium, trace metal and vitamin solutions were added as supplements as previously described^3^. The pre-culture strain was cultivated in the defined minimal medium. When the strain reached the exponential growth phase with OD_600_ 1–2, it was inoculated into a 100 mL shake flask containing 25 mL of the corresponding medium, with an initial OD_600_ of 0.01. The flask was then incubated at 30 °C with agitation at 200 rpm for the duration of the growth test. Samples were periodically collected to measure glucose levels, intermediate metabolites, and OD_600_ until the strains reached the stationary phase. The maximum specific growth rate (μ_max_) was calculated using the natural logarithm of the OD_600_ values, which ranged from 0.1 to 1, plotted against time. These shake flask cultivations were conducted in biological triplicates. For bioreactor cultivations, a defined minimal medium containing 5 g L^−1^ (NH_4_)_2_SO_4_, 3 g L^−1^ KH_2_PO_4_, 0.5 g L^−1^ MgSO_4_·7H_2_O and 2 % glucose was used. Trace metal and vitamin solutions were added to the medium after autoclaving. All bioreactor experiments were conducted in biological triplicates.

For the spotting analysis, synthetic complete (SC) medium was used, consisting of 1.7 g L^−1^ yeast nitrogen base (YNB) without ammonium sulfate and amino acids, 5 g L^−1^ ammonium sulfate, 1.87 g L^−1^ amino acid mixture, and 2 % glucose or ethanol as the carbon source. SC plates containing 10 μM CCCP (sigma) were prepared in advance and stored at 4 °C until use. Strains were initially cultured in SC medium until reaching the exponential growth phase (OD_600_ ≈ 1). Cultures were then serially diluted and spotted onto SC plates containing glucose, ethanol, or glucose supplemented with CCCP, respectively. The spot assay results are described as representative of three independent biological experiments.

To produce free fatty acids in shake flask, 25 mL of minimal medium containing 20 g L^−1^ glucose was used in a 100 mL flask. Additionally, for CEN.PK *113-11C* background strain,40 mg L^−1^ histidine and 60 mg L^−1^ uracil were added to the medium, and for BY4741 background strain,40 mg L^−1^ histidine, 60 mg L^−1^ uracil, 120 mg L^−1^ leucine, and 40 mg L^−1^ methionine were added to the medium. The overnight preculture was inoculated with an initial OD_600_ of 0.1 and cultivated at 200 rpm and 30 °C for 72 h.

### 4.3 Bioreactor cultivation

DasGip Parallel Bioreactor Systems for Microbiology (Eppendorf) was used for bioreactor cultivation. Pre-cultures with an OD_600_ of approximately 1–2 were used to inoculate 500 mL of minimal medium to a starting OD_600_ of 0.01. The temperature was set at 30 °C, the aeration was controlled using a DasGip MX4/4 module and initially provided at 30 L h^−1^ (1VVM). Initial agitation was set to 400 rpm and increased to maximally 800 rpm depending on the dissolved oxygen level, which was maintained above 30 % via stirrer speed and gas flow rate controlled by the DasGip control system. The pH was maintained at 5.0 using 1 M solutions of HCl and 2 M KOH.

### 4.4 Analytical methods

The glucose and metabolites were determined by HPLC. Culture samples were centrifuged at 10,000 g for 1 min, and the supernatants were filtered through a 0.20 μm nitrocellulose filter and analyzed on a HPLC (SHIMADZU, JAPAN) with an Aminex HPX-87H column (Bio-Rad, Hercules, USA) at 65 °C, using 5 mM H_2_SO_4_ as the mobile phase at a flow rate of 0.6 mL min^−1^ for 26 min. Glucose, ethanol and glycerol concentration were detected using a RI-101 Refractive Index Detector. Pyruvate, succinate, and acetate were detected with a DAD-3000 Diode Array Detector at 210 nm (Qin et al., 2020). Samples for determining cell dry weight (DCW) were collected when the culture reached an OD_600_ of approximately 1. DCW was measured gravimetrically using pre-weighed membrane disk filters (PES, 0.45 μm, Pall, USA). For each measurement, 5 mL of culture broth was filtered through the membrane, followed by three washes with 5 mL of deionized water. The filters were then dried at 70 °C for 48 h and weighed again. DCW was calculated based on the weight difference before and after filtration. A biomass composition of CH_1.8_O_0.5_N_0.2_ was assumed (Qin et al., 2024). Biomass yield was determined from samples collected during the mid-exponential growth phase based on optical density at 600 nm and dry cell weight measurements.

### 4.5 FBA

Flux balance analysis (FBA) (Orth et al., 2010) was performed using the *S. cerevisiae* genome-scale metabolic model Yeast8 (Lu et al., 2019), following the COBRA methodology (Heirendt et al., 2019). Experimentally measured parameters were used to constrain the upper and lower bounds of exchange reactions (Table 1), and growth rate was set as the objective function. The sampling method was performed (Haraldsdóttir et al., 2017) for each strain by sampling 10,000 points. The analysis aimed to rationalize the observed carbon flux redistribution rather than to provide fully independent flux predictions.

### 4.6 Free fatty acid extraction and quantification

Free fatty acids were extracted and converted to their methyl esters following a previously reported method, with minor modifications (Zhou et al., 2016). In detail, 100 μL of cell culture was placed into a 2 mL glass vial (Borosilicate type I class A, CNW), to which 10 μL of 40 % tetrabutylammonium hydroxide (sigma) was immediately added. If the titter exceeded 10 mg L^−1^, the sample was diluted 20-fold with water. To this mixture, 200 μL of dichloromethane (sigma) containing 100 mg L^−1^ pentadecanoic acid (sigma) as an internal standard and 200 mM methyl iodide (sigma) as a methyl donor for the generation of fatty acid methyl esters (FAMEs) were added. The mixture was vortexed at 1400 rpm for 30 min and then centrifuged at 5000 g for 3 min. A 100 μL portion of the lower dichloromethane phase was transferred into a new vial and evaporated to dryness. To dissolve the FAMEs for quantification, 100 μL of hexane (sigma) was added.

For FAMEs quantification, a GC-MS (QP2020, Shimadzu, Japan) equipped with a DB-5MS column (30 m × 0.250 mm × 0.25 μm, Agilent) was used. A 1 μL sample was injected with a splitless model at 240 °C. Helium was employed as the carrier gas at a flow rate of 3.0 mL min^−1^. The temperature program was set as follows: the column temperature was initially held at 40 °C for 2 min, then ramped up at 5 °C min^−1^ to 130 °C, followed by a further ramp at 10 °C min^−1^ to 280 °C, which was maintained for 3 min.

The temperatures of the inlet, mass transfer line, and ion source were set at 280 °C, 280 °C, and 230 °C, respectively. Data was collected in full inspection mode (50–650 m/z) and quantified using GC-MS solution 4.4 software.

### 4.7 RNA-seq sample preparation, profling and analysis

The sample for RNA-seq was taken at OD_600_ around 1. 10 mL medium was immediately transferred into 50 mL tube with 30 mL crushed ice and centrifuged for 3 min at 4000 rpm at 2 °C. After removing the supernatant, the pellets were frozen in liquid nitrogen. The sample was then stored at −80 °C before use. RNA extraction and preliminary data analysis were conducted by Annoroad Gene Technology Co, Ltd (Beijing, China). The functional enrichment analysis of KEGG pathways and biological process were performed using the Database for Annotation, Visualization and Integrated Discovery (DAVID).

### 4.8 Statistics

The physiological data presented the mean ± 95 % confidence interval calculated from three biological replicates (n = 3). Other data was presented as mean ± standard deviation (SD) of biological triplicates. Significant comparisons of two groups were indicated in the graphs statistical analysis performed using a two-tailed unpaired Student's *t*-test (∗*p* < 0.05, ∗∗*p* < 0.01, ∗∗∗*p* < 0.001).

## CRediT authorship contribution statement

**Lingyun Li:** Writing – review & editing, Writing – original draft, Project administration, Methodology, Investigation, Data curation. **Xin Chen:** Writing – review & editing, Methodology, Investigation. **Yijie Zhang:** Writing – review & editing, Investigation. **Ning Qin:** Investigation, Conceptualization. **Yu Chen:** Investigation. **Xu Ji:** Investigation. **Jens Nielsen:** Writing – review & editing, Supervision. **Zihe Liu:** Writing – review & editing, Supervision, Funding acquisition, Conceptualization.

## Ethics approval and consent to participate

Not applicable.

## Consent for publication

Not applicable.

## Declaration of competing interest

The authors declare that they have no known competing financial interests or personal relationships that could have appeared to influence the work reported in this paper.

## Data Availability

The RNA-seq raw data can be downloaded from the National Center for Biotechnology information (NCBI, https://www.ncbi.nlm.nih.gov/bioproject/PRJNA1023876). All study data are included in the article and SI Appendix. The materials that were reported in this study are available from the corresponding authors upon reasonable request.
