# Supplementary material for: Mechanistic and applied study of phosphofructokinases, the “gatekeeper” of the glycolytic pathway on the central carbon metabolism
Source: Metab Eng Commun. 2025 Dec 24;22:e00268. doi: 10.1016/j.mec.2025.e00268 (PMC12808500; doi:10.1016/j.mec.2025.e00268)
Supplement: Multimedia component 1 [file mmc1.docx]

**Mechanistic and applied study of phosphofructokinases, the "gatekeeper" of the glycolytic pathway on the central carbon metabolism**

Lingyun Li^1, 2, 5^, Xin Chen^2, 5^, Yijie Zhang^1, 5^, Ning Qin^1^, Yu Chen^3^, Xu Ji^1^, Jens Nielsen^1, 2 ,4^, Zihe Liu^1, *^

This file includes: Supplementary Figure 1-3

Supplementary Table 1-4

^
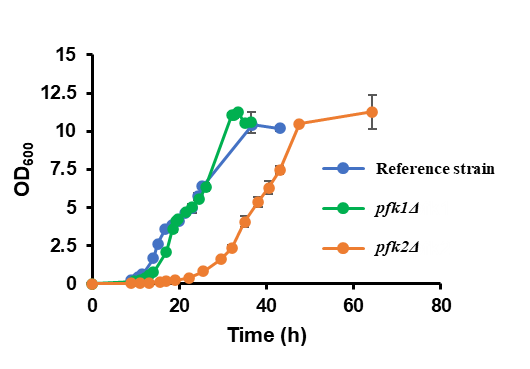
^

**Supplemental Figure 1** Growth curve of *pfk1Δ*, *pfk2Δ*, and reference strain. The data represents three biological replicates

**
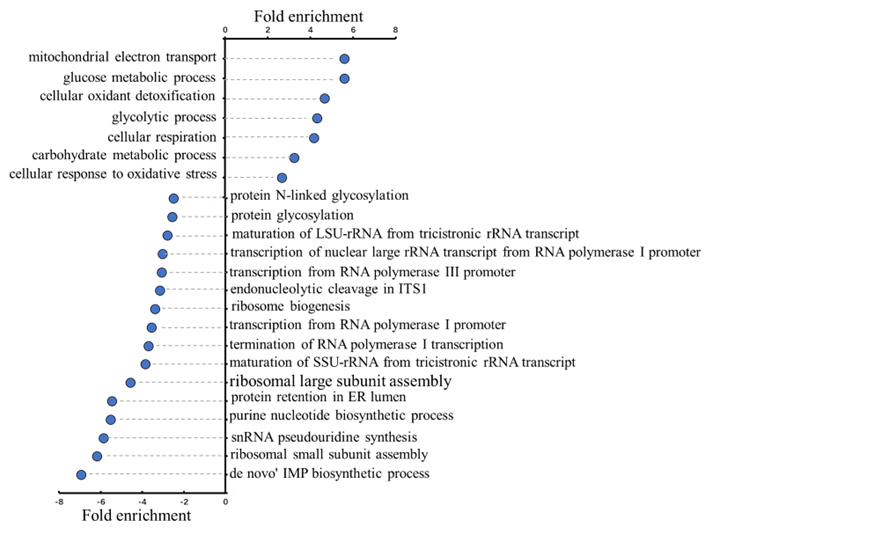
**

**Supplemental Figure 2** Gene set enrichment analysis of significantly differentially expressed genes in the *PFK2* deleted strain compared to reference strain (biological process, FDR < 0.05). Fold enrichment indicated the magnitude of enrichment against the genome background of strain S288C analyzed via DAVID.

**
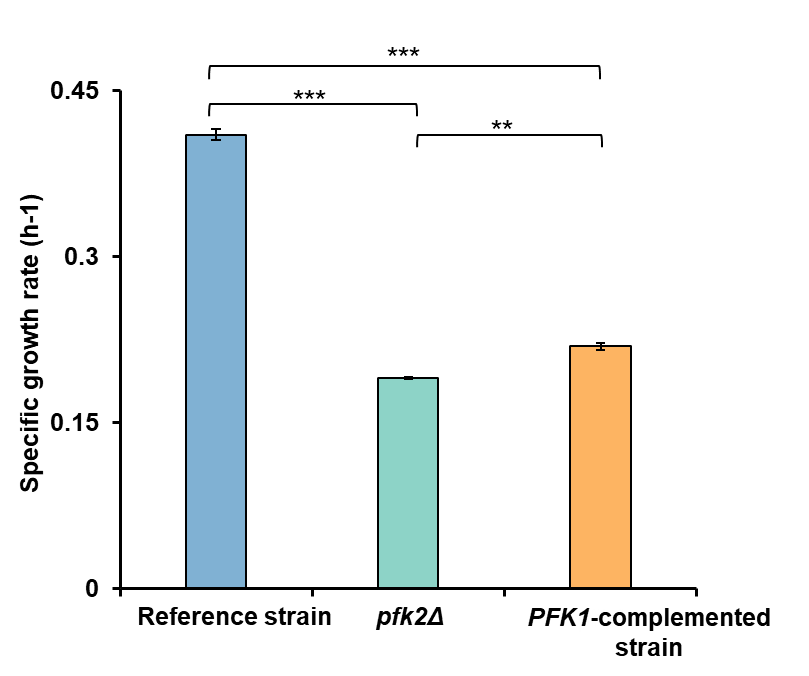
**

**Supplemental Figure 3** Comparison of specific growth rates. The *PFK1*-complemented strain in which an additional copy of *PFK1* was introduced into the *pfk2Δ* background under the control of native *PFK2* promoter. The data represents three biological replicates, and the error bars represent standard deviations. ***p* < 0.01, ****p* < 0.001.

**Supplementary Table 1** Overview of physiological parameters for strains.

|  | **Reference strain** | ***pfk1Δ*** | ***pfk2Δ*** |
| --- | --- | --- | --- |

| Specific growth rate (h^-1^) | 0.41 ± 0.0057 | 0.35 ± 0.0023^***^ | 0.19 ± 0.0011^***^ |
| --- | --- | --- | --- |
| *q*_Glucose_ /μ (mmol g_DW_^-1^) | -46.61 ± 4.41 | -35.43 ± 0.38^*^ | -35.43 ± 1.07^**^ |
| *q*_Glycerol_ /μ _(_mmol g_DW_^-1^) | 6.59 ± 1.11 | 2.83 ± 0.014^*^ | 1.16 ± 0.012^**^ |
| *q*_Ethanol_ /μ _(_mmol g_DW_^-1^) | 69.51 ± 0.42 | 52.11 ± 0.058^***^ | 27.53 ± 1.31^***^ |
| *q*_acetate_ /μ (mmol g_DW_^-1^) | 0.85 ± 0.055 | 1.26 ± 0.031^***^ | 3.32 ± 0.047^***^ |
| *q*_pyruvate_ /μ _(_mmol g_DW_^-1^) | 0.30 ± 0.0023 | 0.289 ± 0.0068^n.s.^ | 0.232 ± 0.0057^***^ |
| Final biomass concentration  (g L^-1^) | 8.66 ± 0.078 | 8.48 ± 0.59 ^n.s.^ | 8.77 ± 1.03 ^n.s.^ |

| Cultivations were carried out in minimal medium containing 2% (w/v) glucose under aerobic conditions. Dissolved oxygen (DO) was controlled at > 30% air saturation by adjusting stirring speed and aeration rate. Values represent mean ± 95% confidence intervals calculated from three biological replicates (n = 3). Statistical significance was assessed using a two-tailed unpaired Student’s t-test (**p* < 0.05, ***p* < 0.01, ****p* < 0.001, n.s., not significant). |
| --- |

**Supplementary Table 2** Plasmids used in this study.

| Plasmids | Relevant characteristics | Reference |
| --- | --- | --- |
| pCas | 2μ, AmpR *TEF1p-iCas9 SNR52p* | (Zhang et al., 2019) |
| pScURA | PCR template for *gRNA-URA3-SNR52p-tGly* | (Zhang et al., 2019) |
| pCas9_FAA1/4/POX1 | ori, AmpR, 2μ, *URA3*, *pTEF1-iCas9-tADH1, pSNR52-*(*FAA1gRNA)-(FAA4gRNA)-(POX1 gRNA)* scaffold-*tSNR52* | (Zhang et al., 2019) |
| pCas9_PFK1 | ori, AmpR, 2μ, *URA3*, *pTEF1-iCas9-tADH1, pSNR52*-*(PFK1 gRNA)-gRNA* scaffold-*tSNR52* | This study |
| pCas9_PFK2 | ori, AmpR, 2μ, *URA3*, *pTEF1-iCas9-tADH1, pSNR52-(PFK2 gRNA)-gRNA* scaffold*-tSNR52* | This study |
| pUG6 | ori, AmpR, 2μ, *pTEF1-kanMX-tTEF1* | (Guldener, 1996) |

**Supplementary Table 3** Strains used in this study.

| Plasmids | Relevant characteristics | | Reference |
| --- | --- | --- | --- |
| CEN.PK 113-7D | Lab strain constructed by Michael Ciriacy and K.D. Entian | (Van Dijken et al., 2000) | |
| CEN.PK 113-11C | *MATa SUC2 MAL2-8c his3Δ1 ura3-52* | (van Dijken et al., 2000） | |
| BY4741 | BY4741 *MATa his3Δ1 leu2Δ0 met15Δ0 ura3Δ0* | (Winston et al., 1995) | |
| CEN.PK 113-7D *pfk1Δ* | CEN.PK 113-7D *pfk1Δ::kanMX* | This study | |
| CEN.PK 113-7D *pfk2Δ* | CEN.PK 113-7D *pfk2Δ::kanMX* | This study | |
| LQ1 | CEN.PK 113-11C *faa1Δ*, *faa4Δ*, *pox1Δ* | This study | |
| LQ2 | CEN.PK 113-11C *faa1Δ*, *faa4Δ*, *pox1Δ* *pfk1Δ* | This study | |
| LQ3 | CEN.PK 113-11C *faa1Δ*, *faa4Δ*, *pox1Δ pfk2Δ* | This study | |
| LQ4 | BY4741 *faa1Δ*, *faa4Δ*, *pox1Δ* | This study | |
| LQ5 | BY4741 *pfk1Δ* | This study | |
| LQ6 | BY4741 *pfk2Δ* | This study | |

**Supplementary Table 4** Primer sequences from this study.

| Primer name | Primer 5’-3’ |
| --- | --- |
| pCAS-PFK1-ORF-guide-F | AAAGGTCTCAGATCAACAACAAAGGCACGGGAGTGTTTTAGAGCTAGAAATAGCAAGTTAAAATAAG |
| PFK1-ORF-donor-F | TATTTGGGAAAGCTTTTATATAAAAAATCTGAAACAAAATCATATCAAAGATGATTGCAA |
| PFK1-ORF-donor-R | ATGCCATTTTTACCTCCTTTTGCTTAACTTAAACTTTTCATTGCAATCATCTTTGATATG |
| kanMX-PFK1-F | GGGAAAGCTTTTATATAAAAAATCTGAAACAAAATCATATCAAAGCAGCTGAAGCTTCGTACGCTGCAGG |
| kanMX-PFK1-R | ATTTTTACCTCCTTTTGCTTAACTTAAACTTTTCATTGCAATCATCGGCCGCATAGGCCACTAGTGGATC |
| pCAS-PFK2-ORF-guide-F | AAAGGTCTCAGATCGATCCATTTGATCTTGCCATTCGCGTTTTAGAGCTAGAAATAGCAAGTTA |
| PFK2-ORF-donor-F | ATAGAACTAGATTTAGAGACTAGTTTAGCATTGGCCAAGAACTAACCATACGCCACTTTC |
| PFK2-ORF-donor-r | GAAAAACATGGGTTAACATTAATTGACATTAATAATAGAAAGTGGCGTATGGTTAGTTC |
| kanMX-PFK2-F | GATTTAGAGACTAGTTTAGCATTGGCCAAGAACTAACCATACGCACAGCTGAAGCTTCGTACGCTGCAGG |
| kanMX-PFK2-R | TAATTGACATTAATAATAGAAAGTGTAATAAAAGGTCATTTTCTTCGGCCGCATAGGCCACTAGTGGATC |
| FAA1-ORF-donor-F | GGATACAATAAAAACTAGAACAAACACAAAAGACAAAAAAAGACAACAATTGGATCAACATTTCCATG |
| FAA1-ORF-donor-R | CTGAAAAAGTGCTTTAGTATGATGAGGCTTTCCTATCATGGAAATGTTGATCCAATTGTTGTCTTTTTTTGTC |
| FAA4-ORF-donor-F | TTTTCTCTGTTCTTCACTATTTCTTGAAAAACTAAGAAGTACGCATCAAAAGGAAGACATAGTT |
| FAA4-ORF-donor-R | ACGTAGTGTTTATGAAGGGCAGGGGGGAAAGTAAAAAACTATGTCTTCCTTTTGATGCGTACTTCTTAG |
| POX1-ORF-donor-F | CTAATAAGTATCACAGAAAAAAAGAAAATATAATAAATTAGTATTGCGATGTAGAGGTTTCCTGT |
| POX1-ORF-donor-R | AAACAAAAGTCGCAAAACAGAGGGTTCGAAGGAAAACAGGAAACCTCTACATCGCAATACTAATT |

**Reference**

Guldener, U., 1996. A new efficient gene disruption cassette for repeated use in budding yeast. Nucleic Acids Res. 24, 2519–2524. https://doi.org/10.1093/nar/24.13.2519

Van Dijken, J.P., Bauer, J., Brambilla, L., Duboc, P., Francois, J.M., Gancedo, C., Giuseppin, M.L.F., Heijnen, J.J., Hoare, M., Lange, H.C., Madden, E.A., Niederberger, P., Nielsen, J., Parrou, J.L., Petit, T., Porro, D., Reuss, M., Van Riel, N., Rizzi, M., Steensma, H.Y., Verrips, C.T., Vindeløv, J., Pronk, J.T., 2000. An interlaboratory comparison of physiological and genetic properties of four Saccharomyces cerevisiae strains. Enzyme Microb. Technol. 26, 706–714. https://doi.org/10.1016/S0141-0229(00)00162-9

Winston, F., Dollard, C., Ricupero‐Hovasse, S.L., 1995. Construction of a set of convenient *Saccharomyces cerevisiae* strains that are isogenic to S288C. Yeast 11, 53–55. https://doi.org/10.1002/yea.320110107

Zhang, Yueping, Wang, J., Wang, Z., Zhang, Yiming, Shi, S., Nielsen, J., Liu, Z., 2019. A gRNA-tRNA array for CRISPR-Cas9 based rapid multiplexed genome editing in *Saccharomyces cerevisiae*. Nat. Commun. 10, 1053. https://doi.org/10.1038/s41467-019-09005-3
